# A Novel Hydrazinecarbothioamide as a Potential Corrosion Inhibitor for Mild Steel in HCl

**DOI:** 10.3390/ma6041420

**Published:** 2013-04-02

**Authors:** Ahmed A. Al-Amiery, Abdul Amir H. Kadhum, Abu Bakar Mohamad, Sutiana Junaedi

**Affiliations:** 1 Department of Chemical & Process Engineering, Universiti of Kebangsaan Malaysia (UKM), Bangi, Selangor 43000, Malaysia; E-Mails: amir@eng.ukm.my (A.A.H.K.); drab@eng.ukm.my ; sutianajnd10@gmail.com (S.J.); 2 Applied Chemistry Division, Applied Science Department, University of Technology (UOT), Baghdad 10001, Iraq

**Keywords:** mild steel, thiosemicarbazide, EIS, HCB, corrosion inhibition

## Abstract

2-(1-methyl-4-((E)-(2-methylbenzylidene)amino)-2-phenyl-1H-pyrazol-3(2H)-ylidene)-hydrazineecarbothioamide (HCB) was synthesized as a corrosion inhibitor from the reaction of 4-aminoantipyrine, thiosemicarbazide and 2-methylbenzaldehyde. The corrosion inhibitory effects of HCB on mild steel in 1.0 M HCl were investigated using potentiodynamic polarization (PDP) and electrochemical impedance spectroscopy (EIS). The results showed that HCB inhibited mild steel corrosion in acidic solution and inhibition efficiency increased with an increase in the concentration of the inhibitor. The inhibition efficiency was up to 96.5% at 5.0 mM. Changes in the impedance parameters suggested that HCB adsorbed on the surface of mild steel, leading to the formation of a protective film. The novel corrosion inhibitor synthesized in the present study was characterized using Fourier transform infrared spectroscopy (FTIR) and nuclear magnetic resonance (NMR) spectral data.

## 1. Introduction

**Figure 1:**
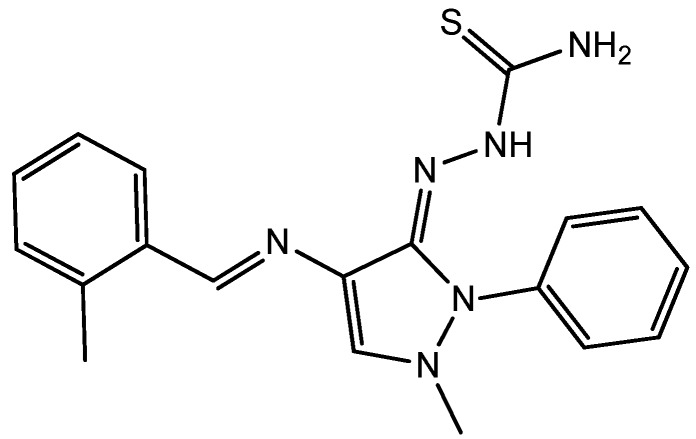
Structure of 2-(1-methyl-4-((E)-(2-methylbenzylidene)amino)-2-phenyl-1H-pyrazol-3(2H)-ylidene)-hydrazineecarbothioamide (HCB).

The corrosion of mild steel is the most common form of corrosion, especially in acidic solution [1]. The majority of well-known inhibitors are organic compounds containing multiple bonds and heteroatoms, such as O, N or S, which allow adsorption onto the metal surface [2]. These compounds can adsorb onto the metal surface and block active surface sites, reducing the rate of corrosion. Organic inhibitors can adsorb onto the metal/solution interface via four distinct mechanisms: (a) electrostatic attraction between charged molecules and the metal; (b) interaction between uncharged electron pairs in the molecule and the metal; (c) interaction between p-electrons and the metal; and (d) a combination of mechanism (a) and (c) [3]. However, the stability of the inhibitor film on the metal surface depends on the physicochemical characteristics of the molecule, which are related to the presence of certain functional groups, aromaticity, steric effects, electronic density of the donors, type of corrosive medium, structure of the inhibitor, charge of the metal surface and nature of the interaction between the p-orbitals of the inhibitor and the d-orbitals of iron [4]. Compounds with functional groups containing heteroatoms, which can donate lone pairs of electrons, are particularly useful as inhibitors for metal corrosion [5,6,7,8]. Moreover, compounds with p-bonds also exhibit good inhibitive properties by providing electrons that interact with the metal surface [9,10,11,12]. The aforementioned features can be combined within the same molecule, as observed in some types of drugs. In recent years, researchers have focused on the development of drugs as inhibitors for metallic corrosion. In the literature, several authors have reported the influence of drugs on the corrosion of metals in acidic media [13,14,15,16]. The study of corrosion processes and their inhibition by organic inhibitors is an active field of research [17]. Many researchers have reported that inhibition mainly depends on the physicochemical and electronic properties of the organic inhibitor, which are related to the presence of certain functional groups, steric effects, electronic density of donor atoms and orbital character of donating electrons, *etc*. [18]. Schiff bases organic compounds with the general formula R–C=N–R-, where R and R- are aryl, alkyl or heterocyclic groups formed by the condensation of a primary amine and a carbonyl group, are potential inhibitors. The greatest advantage of Schiff bases is that they can be conveniently and easily synthesized from relatively cheap starting materials. Due to the presence of imine groups (–C=N–) and electronegative nitrogen, sulfur and/or oxygen atoms in the molecule, Schiff bases are effective inhibitors for the corrosion of steel in acidic media [19,20,21,22]. Moreover, the surface state and excess charge of the metal surface also affect the adsorption behavior of inhibitor molecules on the metal surface [23]. Employing inhibitors is one of the cost-effective protection methods of metals and alloys in acids. Generally, the tendency to form stronger coordination bonds and, as a result, the inhibition efficiency increases according to the following trend: O < N < S < P [24]. The study of organic corrosion inhibitors is an attractive field of research, due to its usefulness in various industries [25]. It has been reported that heterocyclic organic components have better inhibition role in acidic media [26]. In continuation of previous studies [27,28,29,30,31,32,33,34,35,36], we have focused on synthesis of new heterocyclic compounds, and herein, we are reporting the synthesis of, 2-(1-methyl-4-((E)-(2-methylbenzylidene)amino)-2-phenyl-1H-pyrazol-3(2H)-ylidene)hydrazinecarbothio-amide (HCB), as novel organic corrosion inhibitor, and its chemical structure was elucidated using spectroscopic techniques (infrared (IR) and NMR). Recent studies have shown that organic compounds containing polar functions groups and heterocyclic compounds containing polar groups and π-electrons are quite efficient at minimizing the effects of corrosion. The molecular design of *HCB* is based on the fact that thiosemicarbazides, which contain imine, mercapto and sulfide functional groups, and 4-aminoantipyrine, which also possesses imine and methylamine groups and π-electrons bonds, would effectively contribute towards the inhibition of mild steel corrosion in acidic media. In addition, the resonance effect of HCB increases the inhibition activity. The structure of this novel corrosion inhibitor is shown in Figure 1. 

## 2. Results and Discussion

### 2.1. Chemistry

#### Synthetic Chemistry

**Scheme 1:**
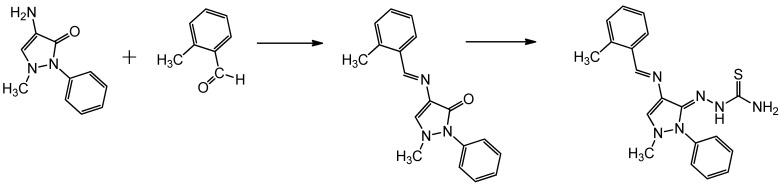
Synthesis of HCB.

For the synthesis of the novel corrosion inhibitor, HCB, the reaction sequences outlined in Scheme 1 were followed. We started from 4-aminoantipyrine, which is commercially available. The synthesis of HCB was achieved by refluxing 4-aminoantipyrine with 2-methylbenzaldehyde in the presence of several drops of acetic acid. The mechanism of this reaction is in accordance with the Schiff base mechanism. The product was refluxed with thiosemicarbazide to produce the target compound in high yield.

**Scheme 2:**
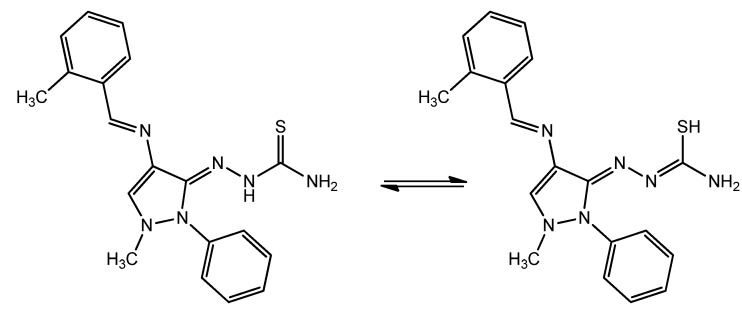
Tautomerization of HCB.

The IR spectrum provides good evidence for the formation of HCB. Namely, the carbonyl group at 1700 cm^−1^ was not observed [37,38], and new bands appeared at 3418.5, 3200.0 and 3159.7 cm^−1^ [39], which are indicative of amino groups. In the IR spectrum of HCB, the stretching frequency of imines was observed at 1615.6 cm^−1^ [40], The high value of the wave number for the C=N group was due to the conjugation (resonance effect) of substituted double bonds, whereas the aromatic carbon-carbon double bond stretch appeared at 1541.3 cm^−1^ [36]. Two types of tautomers, including a thione and thiol or amine and imine, can be expected from HCB (Scheme 2).

In the ^1^H-NMR spectrum of HCB, a doublet of doublets was observed at 6.105 ppm, due to the imine proton, and a doublet was detected at 6.780 ppm, due to the C=C-H proton.

### 2.2. Electrochemical Analysis 

#### Polarization Measurements

The observed changes in the numerical values of the corrosion current density (ICORR), corrosion potential (Ecorr), anodic Tafel slope (βa), cathodic Tafel slope (βc), degree of surface coverage (*θ*) and inhibition efficiency (IE%) due to changes in the concentration of HCB at various temperatures are depicted in Table 1. The surface coverage (*θ*) was calculated as [41]:

*θ = i_corr(uninh)_ − i_corr(inh)_/i_corr(uninh)_* (1)

where icorr(uninh) and icorr(inh) are the corrosion current densities in the absence and presence of inhibitor, respectively. The inhibition efficiency (IE%) can be expressed as:

*IE% = θ ×100 * (2)

**Figure 2:**
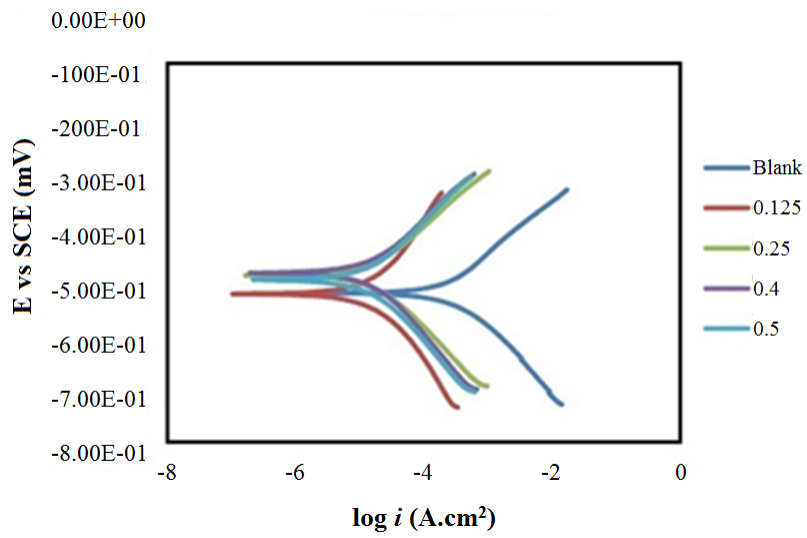
Potentiodynamic polarization curves for mild steel in 1.0 M HCl solution at 30 °C containing various concentrations of HCB.

The results showed that the inhibition efficiency increased with an increase in the concentration of inhibitor. Such behavior indicates that the inhibitor adsorbed onto the metal surface [42]. In acidic media, the anodic reaction of corrosion is the passage of metal ions from the metal surface into solution, and the cathodic reaction is the discharge of hydrogen ions, which produces hydrogen gas or reduces oxygen. The inhibitor may affect either the anodic reaction or the cathodic reaction or both [43]. The anodic Tafel slope (βa) and cathodic Tafel slope (βc) of HCB varied depending on the inhibitor concentration; thus, the inhibitor affected both reactions [44]. HCB can be classified as an anodic- or cathodic-type inhibitor when the change in the Ecorr value is greater than 85 mV [45]. Because the largest displacement exhibited by HCB was 40 mV at 30 °C (Table 1), HCB should be considered a mixed-type inhibitor. In other words, the addition of HCB to a 1.0 M HCl solution reduced the anodic dissolution of mild steel and retarded cathodic hydrogen evolution. The polarization profile of mild steel in 1.0 M HCl at 30 °C in the presence and absence of HCB is shown in Figure 2. The presence of increasing amounts of HCB led to a decrease in both the cathodic and anodic current density. Adsorption is the generally accepted mechanism of the inhibitory effect of organic corrosion inhibitors. The adsorption of inhibitors can alter the rate of corrosion in two different ways: (i) by decreasing the available reaction area, also known as the geometric blocking effect; and (ii) by modifying the activation energies of cathodic and/or anodic reactions that occur in inhibitor-free metal during the inhibited corrosion process. It is difficult to determine which aspects of the inhibiting effect are related to geometric blocking effects and which are related to energy effects. Theoretically, if the geometric blocking effect is stronger than the energy effect, shifts in the Ecorr value should not be observed after the addition of the corrosion inhibitor [41]. 

**Table 1:** Polarization parameters for mild steel in 1.0 M HCl solution at 30°C with various concentrations of HCB*.*

Concentration (mM)	ICORR (μA cm^−2^)	Ecorr(mV *vs*. SCE)	Βa (V dec^−1^)	βc (V dec^−1^)	IE (%)
0	298	504 0	0.119	0.121	0
0.125	24.1	505	0.214	0.193	95.22
0.25	20.4	492	0.180	0.131	95.93
0.4	17.40	466	0.125	0.146	96.26
0.5	16.30	479	0.131	0.141	96.59

IE: Inhibition efficiency

### 2.3. Electrochemical Impedance Spectroscopy (EIS) Measurements

**Figure 3:**
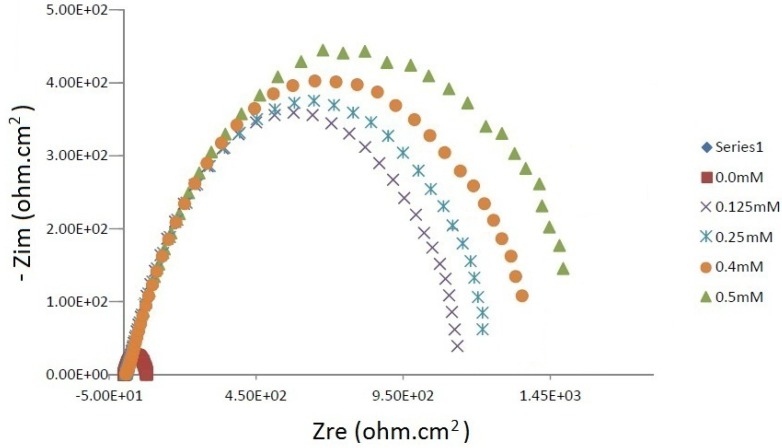
Nyquist plot for mild steel in 1.0 M HCl solutions at 30°C containing various concentrations of HCB.

The experimental results obtained from EIS measurements of the corrosion of mild steel in the presence and absence of inhibitor at 30 °C are summarized in Table 2. The impedance spectra of mild steel in 1.0 M HCl with and without various concentrations of HCB at 30 °C are presented as Nyquist plots in Figure 3. A considerable increase in the total impedance was observed due to the addition of HCB. Thus, as shown in Figure 3, the impedance response of mild steel was significantly changed after the addition of HCB in the corrosive solution, due to an increase in substrate impedance, which was attributed to an increase in the concentration of the inhibitor. In the impedance spectra of mild steel in the absence and presence of HCB, the Nyquist plots displayed two loops, including one loop in the high frequency region (HF) and one loop at an intermediate frequency (MF). Moreover, little inductive behavior was observed at low frequencies (LF). The HF and MF loops were attributed to the limits of the EIS instrument at high frequencies with low resistances and charge-transfer processes, respectively. The inductive behavior observed in the LF region was attributed to the relaxation process of the adsorption of corrosion products or the adsorption of inhibitor molecules on the mild steel surface in acidic solution with and without the inhibitor, respectively [46,47].

**Table 2:** Impedance data for mild steel in 1.0 M HCl solutions with various concentrations of HCB at 30 °C.

Concentration (mM)	Rct (ohm.cm^2^)	Rs (ohm.cm^2^)	Cdl (μFcm^−2^)	IE (%)
0	77.36	1.484	408.1	-
0.125	122.5	7.425	532	39.90
0.25	132.1	7.744	943	94.16
0.4	152.6	7.76	1198	94.89
0.5	168	5.81	1102	96.50

The inhibition efficiencies (IE%) were calculated from the charge transfer resistance using the equation shown below:

(3) $$\text{IE }\left(\text{\%}\right)=\frac{\text{R}\prime \text{ct}-\text{Rct}}{\text{R}\prime \text{ct}}\;\times 100$$

where R^'^_ct_ and R_ct_ indicate the values of the charge transfer resistances in the presence or absence of inhibitor.

It is found that the C_dl_ value decreases from 1198 μF cm^−2^ (0.4 mM) to 1102 μF cm^−2^ (0.5 mM) in the presence of HCB, indicating the thickness of film at 0.5 mM is less than that at 0.4 mM. This proves the hypothesis that the adsorption turns when the concentration of HCB is at 0.5 mM.

**Figure 4:**
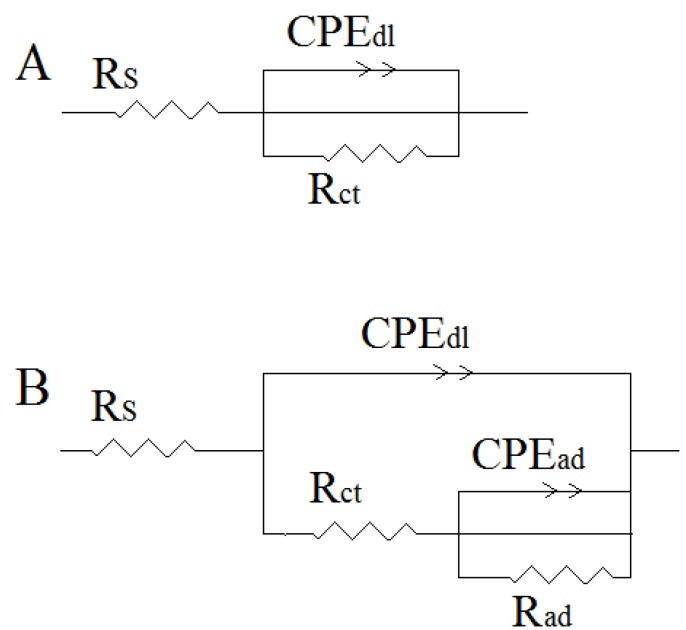
The equivalent circuit model used to fit the impedance data for mild steel in the (**A**) absence; and (**B**) presence of HCB.

Table 2 indicates that the charge-transfer resistance (Rct) increased with an increase in the HCB concentration. Large charge-transfer resistances are associated with systems that corrode slowly [48]. In addition, improved inhibitor protection is associated with a decrease in metal capacitance [49]. The observed decrease in Cdl, which was attributed to a decrease in the local dielectric constant and/or an increase in the thickness of the electrical double layer, suggested that HCB adsorbed to the metal/solution interface [50]. The observed increase in the Cdl value in 1.0 M HCl in the presence of increasing concentrations of HCB may be attributed to a loss of surface heterogeneity, due to inhibitor adsorption on the most active adsorption sites [51]. The corrosion reaction decreased the homogeneity of adsorbed HCB film. In addition, the results showed that the IE% increased with an increase in the concentration of the inhibitor. This result follows the same trend as that of the IE% obtained from the potentiodynamic polarization experiments. Figure 4A,B represent the equivalent circuit design used to fit the experimental EIS data for hydrochloric acid in the presence and absence of the inhibitor. The circuit elements for the data include solution resistance (Rs), a constant phase element (CPEdl) and charge transfer resistance (Rct). The value of Rct was indicative of electron transfer across the interface. Rad in parallel with a CPEad was used as a model for inhibitor adsorption [41]. 

In Figure 4A,B Rs is the solution resistance; Rct is the charge transfer resistance; CPEdl is the constant phase element of the double layer; Rad is the adsorbed layer resistance and CPEad is the constant phase element of the adsorbed layer.

## 3. Experimental Section 

All of the chemicals used in the present study were reagent grade (supplied Sigma-Aldrich, Kuala Lumpur, Malaysia) and were used as supplied without further purification. The FTIR spectra were measured using a Thermo Scientific Model Nicolate 6700 Spectrophotometer. NMR spectra were recorded on a Model AVANCE III 600 MHz spectrometer. 

*Synthesis of Corrosion Inhibitor HCB*: A solution of 2-methylbenzaldehyde (0.4 mmol) in 50 mL of ethanol was refluxed with 4-aminoantipyrine (0.4 mmol) for 5 h. After cooling to room temperature, a solid mass separated from solution and was recrystallized from ethanol to afford the target compound in 87% yield. The product (0.2 mmol) was refluxed with thiosemicarbazide (0.2 mmol) in 40 mL of ethanol for 5 h. After cooling to room temperature, a solid mass separated from the solution and was recrystallized from ethanol to afford the product in a 84% yield. ^1^H-NMR (CDCl_3_): δ 6.105 (dd, 1H, N=CH), δ 6.780 (d, 1H, C=CH), δ 6.281–7.335 (m, 1H, C-H aromatic rings), δ 7.951 (s, 1H) for N-H and δ 7.503 (s, 1H) for N-H; FT-IR: 3418.5, 3200.0, 3159.7 cm^−1^ (N-H, amine), 3052.0 and 3020 cm^−1^ (C-H, aromatic), 2920 and 2911.0 cm^−1^ (C-H, alkane), 1615.6 cm^−1^ (C=N, imine), 1604.3 cm^−1^ (C=N, imine), 1521.3 cm^−1^ (C=C, aromatic); Elemental Analysis: C, analysis calculated for C_12_H_8_N_6_S_4_: C, 62.61%; H, 5.53%; N, 23.06%. Found: C 61.04%, H, 5.12% and N 22.21%.

### Electrochemical Measurements

Mild steel specimens obtained from the Metal Samples Company were used as working electrodes throughout the study. The composition (wt.%) of the mild steel can be described as follows: Fe = 99.21, C = 0.21, Si = 0.38, P = 0.09, S = 0.05 and Al = 0.01 [sample area = 4.5 cm^2^; density = 7.85 g/cm^3^; equivalent wt. = 27.92]. The specimens were cleaned according to the ASTM standard, G1-03 [52]. The measurements were carried out at 30 °C in aerated non-stirred 1.0 M HCl solutions with an HCB concentration range of 1.25 × 10^−4^ to 5 × 10^−4^ M. Solutions were freshly prepared from analytical grade chemical reagents using distilled water. All of the measurements were performed in triplicate, and the average values are reported. The measurements were performed using a Gamry Instrument Potentiostat/Galvanostat/ZRA. DC105 and EIS300 software produced by Gamry were used for potentiodynamic scans and electrochemical impedance spectroscopy (EIS). The potentiodynamic current-potential curves were swept from −0.25 to +0.25 V_sce_ at a scan rate of 0.5 mVs^−1^. All of the impedance data were fitted to appropriate equivalent circuits (EC) using Gamry Echem Analyst software. To stabilize the steady state potential, electrochemical measurements were initiated approximately 30 min after the working electrode was immersed in the solution. 

## 4. Conclusions 

In the present study, 2-(1-methyl-4-((E)-(2-methylbenzylidene)amino)-2-phenyl-1H-pyrazol-3(2H)-ylidene)hydrazinecarbothio-amide (HCB), a novel hydrazinecarbothioamide, was synthesized and characterized using various spectroscopic methods. Changes in the electrochemical impedance spectroscopy (EIS) and potentiodynamic polarization data were used to study the inhibition of mild steel corrosion in 1.0 M HCl solution at 30 °C, and HCB was used as an inhibitor at various concentrations. HCB exhibited excellent inhibition performance as a mixed-type inhibitor. In general, the acidic corrosion of mild steel was reduced upon the addition of an appropriate concentration of HCB. The inhibition efficiencies obtained from EIS data were comparable to those obtained from polarization measurements performed on the inhibitory solution, which were greater than those displayed by the non-inhibitory solution. HCB acted as an efficient corrosion inhibitor in 1.0 M HCl and exhibited a maximum inhibition efficiency of 95%–97%. The adsorption of HCB on mild steel surface obeys the Langmuir adsorption isotherm.
